# Predicting the risk of acute kidney injury in primary care: derivation and validation of STRATIFY-AKI

**DOI:** 10.3399/BJGP.2022.0389

**Published:** 2023-05-03

**Authors:** Constantinos Koshiaris, Lucinda Archer, Sarah Lay-Flurrie, Kym IE Snell, Richard D Riley, Richard Stevens, Amitava Banerjee, Juliet A Usher-Smith, Andrew Clegg, Rupert A Payne, Margaret Ogden, FD Richard Hobbs, Richard J McManus, James P Sheppard

**Affiliations:** Nuffield Department of Primary Care Health Sciences, University of Oxford, Oxford.; School of Medicine, Keele University, Keele; Institute of Applied Health Research, University of Birmingham, Birmingham.; Nuffield Department of Primary Care Health Sciences, University of Oxford, Oxford.; School of Medicine, Keele University, Keele.; Institute of Applied Health Research, University of Birmingham, Birmingham.; Nuffield Department of Primary Care Health Sciences, University of Oxford, Oxford.; Institute of Health Informatics, University College London, London.; Primary Care Unit, Department of Public Health and Primary Care, University of Cambridge, Cambridge.; Academic Unit for Ageing and Stroke Research, Bradford Institute for Health Research, University of Leeds, Leeds.; Centre for Academic Primary Care, Population Health Sciences, University of Bristol, Bristol.; Nuffield Department of Primary Care Health Sciences, University of Oxford, Oxford.; Nuffield Department of Primary Care Health Sciences, University of Oxford, Oxford.; Nuffield Department of Primary Care Health Sciences, University of Oxford, Oxford.

**Keywords:** blood pressure, drug-related side effects and adverse reactions, electronic health records, epidemiology, primary health care, vascular diseases

## Abstract

**Background:**

Antihypertensives reduce the risk of cardiovascular disease but are also associated with harms including acute kidney injury (AKI). Few data exist to guide clinical decision making regarding these risks.

**Aim:**

To develop a prediction model estimating the risk of AKI in people potentially indicated for antihypertensive treatment.

**Design and setting:**

Observational cohort study using routine primary care data from the Clinical Practice Research Datalink (CPRD) in England.

**Method:**

People aged ≥40 years, with at least one blood pressure measurement between 130 mmHg and 179 mmHg were included. Outcomes were admission to hospital or death with AKI within 1, 5, and 10 years. The model was derived with data from CPRD GOLD (*n* = 1 772 618), using a Fine–Gray competing risks approach, with subsequent recalibration using pseudo-values. External validation used data from CPRD Aurum (*n* = 3 805 322).

**Results:**

The mean age of participants was 59.4 years and 52% were female. The final model consisted of 27 predictors and showed good discrimination at 1, 5, and 10 years (C-statistic for 10-year risk 0.821, 95% confidence interval [CI] = 0.818 to 0.823). There was some overprediction at the highest predicted probabilities (ratio of observed to expected event probability for 10-year risk 0.633, 95% CI = 0.621 to 0.645), affecting patients with the highest risk. Most patients (>95%) had a low 1- to 5-year risk of AKI, and at 10 years only 0.1% of the population had a high AKI and low CVD risk.

**Conclusion:**

This clinical prediction model enables GPs to accurately identify patients at high risk of AKI, which will aid treatment decisions. As the vast majority of patients were at low risk, such a model may provide useful reassurance that most antihypertensive treatment is safe and appropriate while flagging the few for whom this is not the case.

## INTRODUCTION

Blood pressure lowering (antihypertensive) medications are one of the most commonly prescribed medications in older people.^1^ They are highly effective at reducing the risk of cardiovascular disease (CVD) and mortality;^2^ however, they are also associated with adverse events, including acute kidney injury (AKI), electrolyte abnormalities, hypotension, and syncope.^3^

At present, decisions about when to start (or continue) antihypertensive therapy are made almost exclusively on the basis of blood pressure level and CVD risk, aided by CVD risk prediction models.^4^ In contrast, less emphasis is given to the potential for harm from treatment. To make such informed clinical decisions, GPs need to understand both the effect of treatment on adverse events (which has been shown previously),^3^ and an individual’s underlying risk of harm, which currently remains largely unknown.

One such adverse event is AKI, which is typically defined as an increase in serum creatinine of ≥0.3 mg/dl within the past 48 h or an increase of ≥1.5 times the baseline value within the past 7 days.^5^ Over the past decade, automatic reporting of potential AKI on renal function test reports has become usual practice^6^ and may lead to GPs modifying potentially beneficial treatment. In serious cases, AKI can lead to admission to hospital and reduced quality of life, and acute renal failure, as AKI was previously known, is still a significant and long-standing issue.^7^

Better understanding of an individual’s risk of serious AKI (resulting in admission to hospital or death), along with other adverse events, could better inform GPs making antihypertensive treatment decisions, particularly where such a risk is high. This study therefore aimed to use routinely available data from clinical records to develop and externally validate a clinical prediction model to predict an individual’s underlying risk of experiencing admission to hospital or death with AKI within the next 1, 5, and 10 years, for patients with an indication for antihypertensive treatment.

**Table:** How this fits in

Acute kidney injury (AKI) is one of the more serious adverse events associated with antihypertensive treatment, reducing an individual’s health-related quality of life and increasing the risk of admission to hospital. Clinical guidelines recommend that when prescribing antihypertensives GPs should take into account the likelihood of both the benefits and harms from treatment, but few data exist in regard to the risk of AKI. A clinical prediction model was developed and externally validated for the risk of AKI up to 10 years in the future in patients eligible for antihypertensive medication, incorporating commonly recorded patient characteristics, comorbidities, and prescribed medications. The model showed good discrimination and good calibration for probabilities up to 20%, enabling GPs to accurately identify patients at higher risk of AKI. This could be useful to reassure the majority of patients starting or continuing treatment that their risk of AKI is very low.

## METHOD

Extended methods for this study are described in Supplementary Appendix S1. This study used an observational cohort design and aimed to develop and validate a prediction model for admission to hospital or death with AKI. As a prediction modelling study it was not the aim to examine the association between antihypertensive treatment and AKI, which has been studied previously.^3^ The current study used routine primary care data from the Clinical Practice Research Datalink (CPRD) in England, linked to Office for National Statistics (ONS) mortality data, basic inpatient Hospital Episode Statistics (HES) data, and patient-level Index of Multiple Deprivation (IMD) data. The model was derived using population data from CPRD GOLD and externally validated using CPRD Aurum, each of which is based on data from English general practices using different electronic health record (EHR) software.

### Population

Patients were eligible for this study if they were registered at linked general practices contributing to the CPRD GOLD or Aurum in England. Individual records were included if they related to patients aged ≥40 years, registered to a CPRD ‘up-to-standard’ practice, and had records available after the study start date (1 January 1998). The study end date was 31 December 2018. Patients entered the cohort following their first systolic blood pressure reading ≥130 mmHg, chosen as a group likely to be considered for antihypertensive therapy.^8^ Patients were excluded if they had no record of blood pressure measurement or a systolic blood pressure ≥180 mmHg, as at this level treatment would be indicated regardless of risk of adverse events. The index date was defined 12 months after the patient was recorded as having a systolic blood pressure reading ≥130 mmHg. Patients experiencing AKI on their index date were excluded from the analysis.

### Outcomes

The model outcome was defined as first admission to hospital or death with a primary diagnostic code for AKI within 10 years of the index date. This was based on ICD-9/10 codes documented in HES and ONS mortality data (codes are available in Supplementary Box S1).

### Model predictors

Potential predictors of AKI were identified from the literature^9^^,^^10^ and by expert clinical opinion. These included patient demographics, clinical characteristics, previous conditions, and other prescribed medications (see Supplementary Box S2). Predictors were defined as the most recent relevant clinical code before the index date. Antihypertensive medications were defined as a prescription in the 12 months before the index date.

### Sample size calculation

Assuming a conservative event rate of 24.6 per 100 000 person–years,^11^ an expected median follow-up of 7 years,^12^ an estimate of Nagelkerke’s *R*^2^ statistic of 0.15, and a maximum number of 40 parameters in the model, a sample size of approximately 80 000 patients was estimated to be required for the development of this risk equation.^13^

### Model development

A multivariable model was fitted using a Fine–Gray subdistribution model that takes into account competing risks to avoid overestimation of predicted probabilities.^14^ Deaths from causes other than AKI were treated as a competing event. Automated variable selection methods were not used, as all the variables were predetermined based on the literature and expert clinical opinion. Predictor effects in the model were reported as subdistribution hazard ratios (SHRs) with 95% confidence intervals (CIs), and post-estimation of the baseline cumulative incidence for AKI was calculated using the Breslow estimator as defined in the Fine and Gray article.^14^ Analyses were undertaken using the fastcmprsk package in R (version 4.1.0).

Fractional polynomials were used to identify the optimal functional form of continuous variables. The baseline cumulative incidence function at 1, 5, and 10 years was estimated in the derivation dataset to allow individual risk predictions at these time points.

Initial model calibration was assessed in the development dataset using calibration curves generated from pseudo-values: jack-knife estimators representing an individual’s contribution to the cumulative incidence function for AKI accounting for competing risk, and calculated by the Aalen– Johansen method. These were generated separately in 50 groups by linear predictor value, accounting for the competing risk of death.^15^ Where calibration was observed to be suboptimal at 5 and 10 years, the model in the development data was recalibrated by fitting a generalised linear model (with logit link function) directly to the pseudo-values, with the linear predictor from the Fine–Gray model as the only variable, and allowing for a non-linear recalibration effect using fractional polynomials.

### Missing data

Multiple imputation was used to impute all variables with missing data, separately for each of the development and validation datasets. Ten imputations were generated for each dataset. Imputation models contained all predictors included in the main analysis, as well as the Nelson–Aalen estimator and the outcomes of interest (AKI and death).^16^ The model coefficients and performance measures (such as C-statistic) were estimated from each imputation dataset and combined using Rubin’s rules.^17^

### External validation

The external validation was conducted independently by researchers at a different institution. The final model equation (recalibrated at 5 and 10 years; see Supplementary Box S3) was applied to each individual in the validation cohort to give the predicted probabilities of AKI at 1, 5, and 10 years, while taking into account the competing risk of death.^18^

Model performance was determined using Royston and Sauerbrei’s ${\text{R}}_{D}^{2}$, a truncated C-statistic and the D-statistic.^19^ Model calibration was assessed through comparison of predicted probabilities with observed pseudo-values estimated using jack-knife estimators representing an individual’s contribution to the cumulative incidence function for AKI, accounting for competing risks, and calculated by the Aalen–Johansen method, in the external validation cohort. Calibration was presented as the ratio of observed to expected event probabilities and calibration plots to compare the observed versus predicted risks at 1, 5, and 10 years. A random effects meta-analysis was used to examine heterogeneity in model performance across different GP practices, where case mix and outcome prevalence were expected to vary.

The clinical utility of the model was assessed by plotting the 1, 5, and 10-year risk of AKI against the 10-year risk of CVD, calculated using the QRisk2 algorithm.^4^ A net-benefit analysis was also conducted, where the harms and benefits of using the model to guide treatment/management decisions were compared with either not taking any action for everyone (irrespective of AKI risk) or taking action for everyone.^20^

## RESULTS

### Population characteristics

The CPRD GOLD derivation cohort included 1 772 618 patients with a mean age of 59.4 years (SD 13.2), including 921 867 females (52%) (Table 1 and Supplementary Figure S1). The 10-year prevalence of significant AKI following the index date was 3% (*n* = 56 110) with 10% (*n* = 171 018) of patients experiencing the competing event of death from other causes. Median follow-up time for the cohort was 6.4 years (interquartile range [IQR] 2.7–10.0).

**Table 1.** Descriptive patient statistics

**Patient characteristics**	**GOLD derivation dataset (*n* = 1 772 618), *n* (%)^a^**	**Aurum validation dataset (*n* = 3 805 322), *n* (%)^a^**
**Follow-up, years, median (IQR)**	6.4 (2.7–10.0)	6.9 (2.8–10.0)

**Age, years, mean (SD)**	59.4 (13.2)	58.6 (13.3)

**Sex, female**	921 867 (52)	1 959 472 (51)

**Systolic blood pressure, mean (SD)**	143.5 (11.9)	143.8 (12.3)

**Diastolic blood pressure, mean (SD)**	83.8 (9.6)	83.9 (9.8)

**Total cholesterol, mean (SD)**	5.3 (1.1)	5.5 (1.2)
Missing	868 468 (49)	1 839 103 (48)

**Body mass index**		
Underweight	20 625 (1)	39 987 (1)
Normal	519 374 (29)	1 033 529 (27)
Overweight	586 325 (33)	1 231 156 (32)
Obese	340 241 (19)	757 111 (20)
Morbidly obese	39 831 (2)	95 006 (2)
Missing	266 222 (15)	648 533 (17)

**Ethnicity**		
White	734 167 (41)	2 041 469 (54)
Black	10 799 (1)	115 276 (3)
Asian (South)	14 799 (1)	94 483 (2)
Other	15 732 (1)	832 614 (22)

**Deprivation score, IMD quintile**		
1	420 765 (24)	790 303 (21)
2	406 779 (23)	732 240 (19)
3	376 770 (21)	684 279 (18)
4	313 605 (18)	630 472 (17)
5	254 699 (14)	597 169 (16)
Missing	NA	370 859 (10)

**Smoking status**		
Non-smoker	847 217 (48)	1 475 689 (39)
Ex-smoker	471 008 (27)	1 236 048 (32)
Smoker	363 443 (21)	838 395 (22)
Missing	90 950 (5)	255 190 (7)

**Electronic frailty index, continuous, median (IQR)**	0.03 (0 to 0.08)	0.06 (0.03 to 0.08)

**Alcohol^b^**		
Non-drinker	289 472 (16)	864 849 (23)
Trivial	488 292 (28)	998 943 (26)
Light	239 734 (14)	696 364 (18)
Moderate	179 100 (10)	246 466 (6)
Heavy	22 763 (1)	74 004 (2)
Unknown amount	291 651 (16)	237 458 (6)
Missing	261 606 (15)	687 238 (18)

**Risk factors**		
Chronic kidney disease	37 385 (2)	98 170 (3)
Hypotension/syncope	68 517 (4)	147 533 (4)
Ischaemic heart disease	343 677 (19)	508 226 (13)
Diabetes	137 763 (8)	324 163 (9)
Atrial fibrillation	51 378 (3)	115 266 (3)

**Antihypertensive medications**		
ACE Inhibitors	219 514 (12)	478 763 (13)
Angiotensin II receptor blockers	59 077 (3)	136 917 (4)
Alpha-blockers	34 335 (2)	68 129 (2)
Beta-blockers	216 124 (12)	461 318 (12)
Calcium channel blockers	193 142 (11)	426 141 (11)
Thiazide and thiazide-like diuretics	180 071 (10)	397 971 (10)
Other antihypertensives	10 785 (1)	19 233 (1)
Any antihypertensive medication	556 978 (31)	1 261 268 (33)

a *Unless otherwise stated.*

b *Non drinker (0 units per day), trivial drinker (<1 unit per day), light (1–2 units per day), moderate (2–6 units per day), and heavy (>6 units per day). ACE = angiotensin-converting enzyme. IQR = interquartile range. IMD = Index of Multiple Deprivation. NA = not applicable. SD = standard deviation.*

The CPRD Aurum validation cohort contained 3 805 322 patients, with 131 584 (3%) experiencing admission to hospital or death with AKI during 10-year follow-up (incidence by practice shown in Supplementary Figure S2). The competing event of death affected 407 857 (11%) patients during follow-up (data not shown in Tables or Figures). Median follow-up time in the validation cohort was 6.9 years (IQR 2.8– 10.0) (Table 1).

### Model derivation

The final model included 27 predictors, with transformations used for diastolic blood pressure and total cholesterol because of non-linear relationships with the outcome. Being male, morbidly obese, a smoker, a heavy drinker, more deprived, increasing age or frailty, or a history of chronic kidney disease and diabetes were associated with an increased risk of AKI. Most antihypertensive medications, with the exception of thiazide and thiazide-like diuretics, increased the risk of AKI, with angiotensin-converting enzyme inhibitors (SHR 1.54, 95% CI = 1.51 to 1.57) and angiotensin II receptor blockers (SHR 1.43, 95% CI = 1.38 to 1.48) conferring the highest risk (Table 2).

**Table 2.** Subdistribution hazard ratios (SHR) for covariates included in the final clinical prediction model for AKI within 10 years^a^

**Covariates**	**Full case analysis (*n* = 337 733), SHR (95% CI)**	**Multiple imputation model (*n* = 1 772 618), SHR (95% CI)**
**Age, years, per year increase**	1.04 (1.038 to 1.041)	1.061 (1.060 to 1.062)

**Sex, female**	0.66 (0.64 to 0.68)	0.61 (0.60 to 0.63)

**Systolic blood pressure, per 1 mmHg increase**	1.004 (1.003 to 1.005)	1.005 (1.004 to 1.006)

**Diastolic blood pressure^a^**	1.36 (1.28 to 1.46)	1.29 (1.23 to 1.35)

**Cholesterol^a^**	0.77 (0.72 to 0.82)	0.76 (0.73 to 0.81)

**Body mass index**		
Underweight	Reference	Reference
Normal	0.95 (0.85 to 1.06)	0.97 (0.82 to 1.14)
Overweight	0.95 (0.85 to 1.07)	1.06 (0.89 to 1.26)
Obese	1.16 (1.03 to 1.30)	1.34 (1.11 to 1.62)
Morbidly obese	1.88 (1.67 to 2.12)	2.50 (2.20 to 2.90)

**Ethnicity**		
White	Reference	Reference
Black	1.23 (1.13 to 1.35)	1.33 (1.10 to 1.63)
Asian (South)	1.00 (0.92 to 1.09)	1.18 (0.95 to 1.45)
Other	0.94 (0.87 to 1.02)	1.02 (0.91 to 1.16)

**Deprivation score, IMD quintile**		
1	Reference	Reference
2	1.04 (0.99 to 1.09)	1.08 (1.04 to 1.10)
3	1.08 (1.03 to 1.13)	1.10 (1.07 to 1.14)
4	1.18 (1.13 to 1.23)	1.22 (1.19 to 1.26)
5	1.31 (1.25 to 1.37)	1.37 (1.33 to 1.41)

**Smoking status**		
Non-smoker	Reference	Reference
Ex-smoker	1.17 (1.14 to 1.21)	1.21 (1.18 to 1.23)
Smoker	1.51 (1.45 to 1.56)	1.57 (1.52 to 1.61)

**Electronic frailty index, for every 3.6 deficits^b^**	1.10 (1.07 to 1.20)	1.28 (1.26 to 1.31)

**Alcohol**		
Non-drinker	Reference	Reference
Trivial	0.82 (0.79 to 0.85)	0.85 (0.83 to 0.89)
Light	0.81 (0.78 to 0.84)	0.80 (0.76 to 0.85)
Moderate	0.84 (0.80 to 0.89)	0.82 (0.78 to 0.87)
Heavy	1.15 (1.04 to 1.27)	1.27 (1.14 to 1.41)
Unknown amount	0.91 (0.87 to 0.95)	0.88 (0.84 to 0.93)

**Risk factors**		
Chronic kidney disease	2.10 (2.05 to 2.20)	2.05 (1.98 to 2.12)
Hypotension/syncope	0.86 (0.81 to 0.91)	0.87 (0.84 to 0.91)
Ischaemic heart disease	0.93 (0.90 to 0.96)	0.95 (0.93 to 0.97)
Diabetes	1.15 (1.12 to 1.19)	1.53 (1.49 to 1.57)
Atrial fibrillation	1.27 (1.21 to 1.34)	1.19 (1.16 to 1.23)

**Medications**		
ACE Inhibitors	1.44 (1.40 to 1.49)	1.54 (1.51 to 1.57)
Angiotensin II receptor blockers	1.38 (1.32 to 1.44)	1.43 (1.38 to 1.48)
Alpha-blockers	1.19 (1.13 to 1.25)	1.23 (1.19 to 1.28)
Beta-blockers	1.07 (1.04 to 1.11)	1.16 (1.13 to 1.19)
Calcium channel blockers	1.13 (1.10 to 1.17)	1.19 (1.16 to 1.21)
Thiazide and thiazide-like diuretics	0.92 (0.89 to 0.95)	0.98 (0.95 to 0.99)
Other antihypertensives	1.25 (1.11 to 1.41)	1.27 (1.17 to 1.37)
Opioids	1.10 (1.07 to 1.41)	1.16 (1.13 to 1.18)
Hypnotics/Benzodiazepines	1.05 (1.02 to 1.09)	1.04 (1.02 to 1.06)
Antidepressants	1.04 (0.99 to 1.08)	1.06 (1.04 to 1.08)
Anticholinergic medications	1.03 (0.99 to 1.07)	1.03 (1.00 to 1.06)

a *The following transformations were applied to specific variables in the model: diastolic blood pressure was transformed using a first-degree polynomial (^–0.5), rescaled, and centred ([diastolic blood pressure/1000]^–0.5) – 3.472411. Age was centred = age – 59.40057. Systolic blood pressure was centred = systolic blood pressure – 143.47. The electronic frailty index was rescaled = (frailty index/0.1). Cholesterol was log transformed and centred = ln(cholesterol) – 1.670416.*

b *SHR per deficit: 1.072 (95% CI = 1.068 to 1.076).*

*ACE = angiotensin-converting enzyme. AKI = acute kidney injury. IMD = Index of Multiple Deprivation. SHR = subdistribution hazard ratio.*

### External validation

The distribution of the linear predictor in the validation dataset, grouped by outcome type, can be seen in Supplementary Figure S3. External validation of the model showed good discrimination, with a truncated C-statistic of 0.864 (95% CI = 0.857 to 0.870) at 1 year, 0.838 (95% CI = 0.835 to 0.840) at 5 years, and 0.821 (95% CI = 0.818 to 0.823) at 10 years (Table 3).

**Table 3.** Predictive performance statistics of the models on external validation in the Clinical Practice Research Database (CPRD) Aurum

**Performance statistic**	**Model timeframe**		

	**1-year estimate (95% CI)**	**5-year estimate, recalibrated model (95% CI)^a^**	**10-year estimate, recalibrated model (95% CI)^a^**
**O/E**			
Pooled effect size	0.509 (0.493 to 0.526)	0.685 (0.671 to 0.698)	0.633 (0.621 to 0.645)
Prediction interval	(0.225 to 1.150)	(0.415 to 1.139)	(0.391 to 1.020)
*I*^2^, %	100 (100 to 100)	100 (100 to 100)	100 (100 to 100)
Tau^2^	0.170 (0.153 to 0.190)	0.066 (0.059 to 0.074)	0.060 (0.054 to 0.067)

**C-statistic**			
Pooled effect size	0.864 (0.857 to 0.870)	0.838 (0.835 to 0.840)	0.821 (0.818 to 0.823)
Prediction interval	(0.666 to 0.953)	(0.796 to 0.872)	(0.777 to 0.858)
*I*^2^, %	78.9 (76.3 to 81.3)	38.7 (31.4 to 45.5)	51.4 (45.4 to 57.0)
Tau^2^	0.346 (0.298 to 0.403)	0.020 (0.015 to 0.027)	0.020 (0.015 to 0.025)

**D-statistic**			
Pooled effect size	1.85 (1.69 to 2.01)	1.84 (1.69 to 1.99)	1.55 (1.42 to 1.67)
Prediction interval	(0.91 to 2.78)	(1.23 to 2.44)	(1.30 to 1.79)
*I*^2^, %	7.2 (4.5 to 10.7)	2.6 (1.2 to 4.6)	0.5 (0.0 to 2.3)
Tau^2^	0.220 (0.136 to 0.342)	0.090 (0.041 to 0.164)	0.012 (0.000 to 0.060)

**Royston and Sauerbrei’s** ${\text{R}}_{\text{D}}^{2}$			
Range	0.000 to 1.000	0.011 to 0.769	0.018 to 0.737
Median (IQR)	0.569 (0.479–0.641)	0.492 (0.436–0.549)	0.409 (0.347–0.477)
Mean (SD)	0.540 (0.157)	0.492 (0.087)	0.413 (0.092)

a *Predictive performance of the models that had been recalibrated to the development data, when applied in the external validation data. Performance statistics for the original models can be found in Supplementary Table S3. IQR = interquartile range. O/E = ratio of observed to expected event probabilities. SD = standard deviation.*

**Figure 1.**
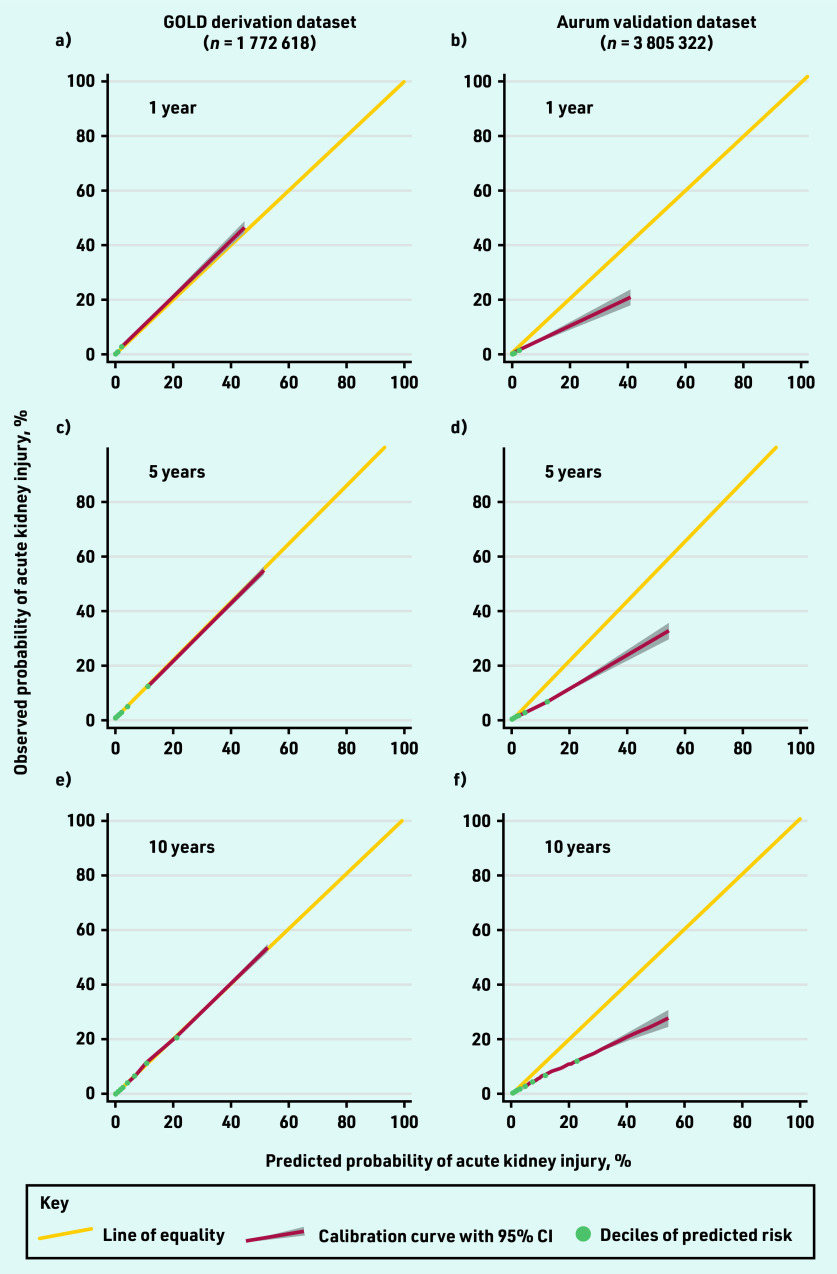
*Calibration plots comparing observed and predicted risk of acute kidney injury at: a and b) 1; c and d) 5; and e and f) 10 years in the GOLD derivation dataset and Aurum validation dataset.*

There was some evidence of model overprediction at each time point, although this was less pronounced in the models that had been recalibrated to the development data at 5 and 10 years (Table 3, Figure 1, Supplementary Figure S4, and Supplementary Table S1). Miscalibration was mostly evident in a small number of patients at higher predicted probabilities (>20% risk).

**Figure 2.**
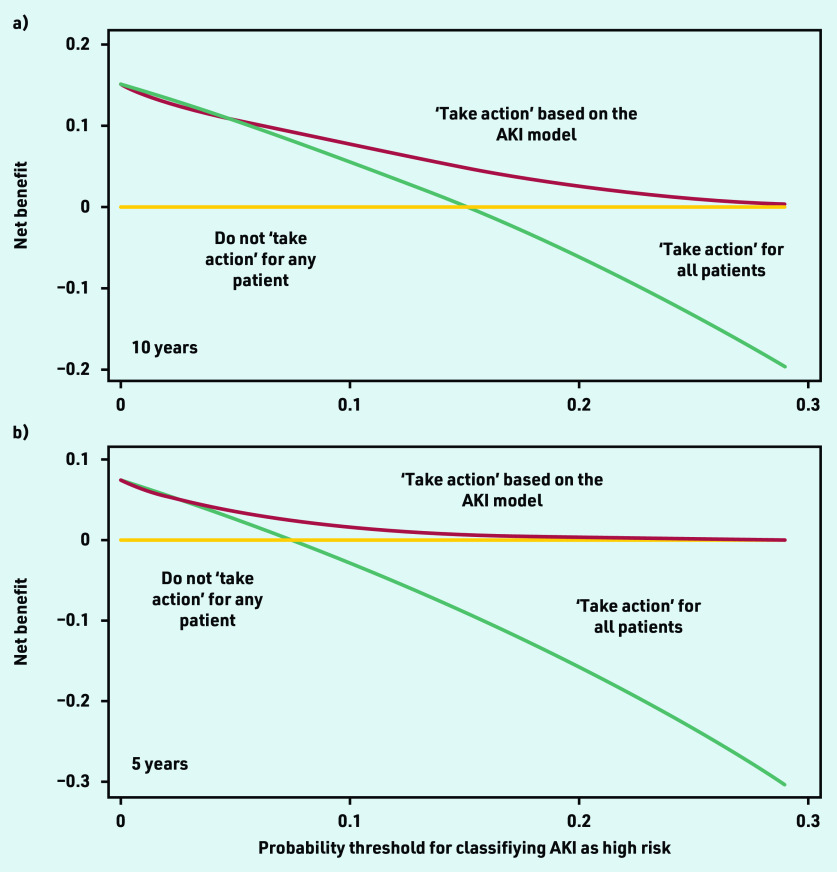
*Decision curve analysis, showing net benefit of using the AKI prediction model for determining which patients are at high risk of AKI at: a) 10 years; and b) 5 years. The y-axis corresponds to the unit of measurement of net benefit (true positives; a net benefit of 0.1 means 10 true positives per 100 patients). The x-axis corresponds to a potential threshold probability from the AKI model (for example, a 10% threshold used to define patients at high risk for developing AKI). All plots have lines corresponding to the net benefit of ‘taking action’ to address the high AKI risk (either through monitoring, or deprescribing of antihypertensive drugs). Treat all corresponds to ‘taking action’ for all patients irrespective of AKI risk. Treat none corresponds to not ‘taking action’ for anyone. The line that is the highest corresponds to the best strategy at any given threshold probability. AKI = acute kidney injury.*

Net-benefit analysis showed that using the model with an AKI risk threshold of ≥10% to define those at high risk (potentially requiring action), would result in higher clinical utility compared with other approaches, such as assuming that everyone is at high risk of AKI or that all patients have low risk (Figure 2). Model performance varied more among smaller practices, with more consistent performance seen as practice size increased (see Supplementary Figure S5).

**Figure 3.**
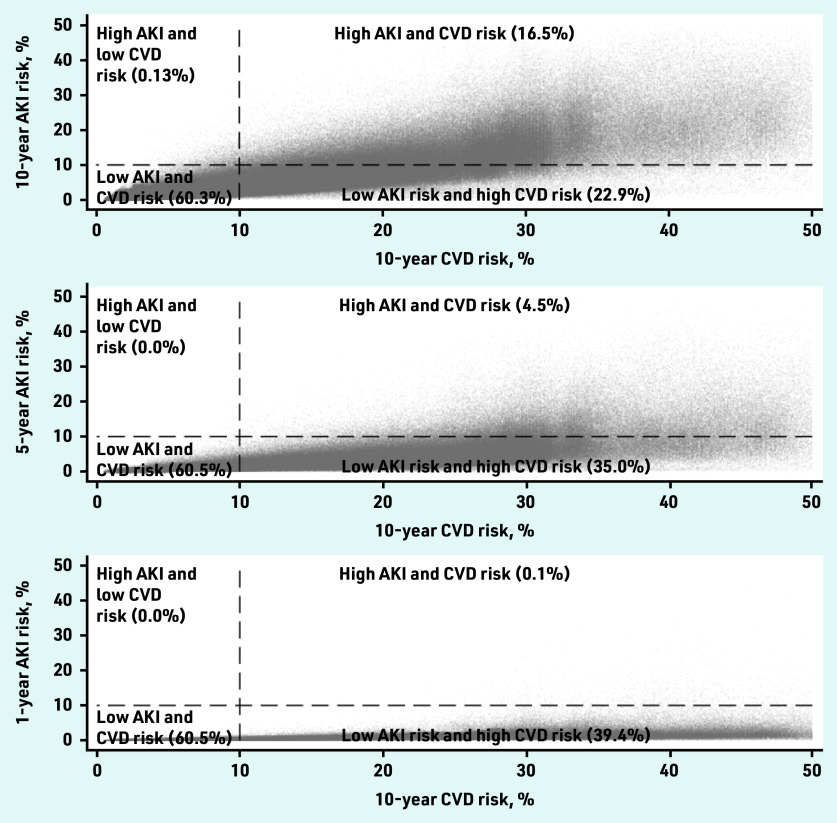
*Comparison of patients’ risk of cardiovascular disease (QRisk2) and AKI at: a) 10 years; b) 5 years; and c) 1 year in the GOLD derivation dataset. AKI = acute kidney injury. CVD = cardiovascular disease.*

Overall, most patients had a low risk of AKI, with 1 770 999 patients (99.9%) estimated to have a <10% 1-year risk; 1 693 695 (96%) estimated to have a <10% 5-year risk; and 1 477 166 (83%) estimated to have a <10% 10-year risk. Only 2677 patients (0.1%) had a high risk of AKI (>10%) but low risk of CVD (<10%) (Figure 3 and Supplementary Table S2). There was a higher prevalence of obesity, deprivation, and prescription of antihypertensive medications in this group (see Supplementary Table S3).

## DISCUSSION

### Summary

In this study, a clinical prediction model to identify those more at risk of AKI leading to significant harm within 10 years in patients with an indication for antihypertensive treatment showed that most had very-low risk, particularly in the medium term (≤5 years). The model incorporated commonly recorded patient characteristics, comorbidities, and prescribed medications, and showed good discrimination on external validation. Where miscalibration occurred this primarily affected the small proportion of patients with a very-high risk of AKI (>20% over 10 years, *n* = 204 775 patients [5%]).

Such a tool could therefore be useful for GPs and pharmacists to reassure most patients that their risk of AKI is low, and although treatment with medications such as antihypertensives might increase this risk^3^ it is unlikely to outweigh the potential benefits from reducing blood pressure and CVD risk. For the small number where this is not the case, the tool could flag this to allow incorporation into clinical decision making.

### Strengths and limitations

This study used two large, population-based cohorts to derive and externally validate a clinical prediction model for admission to hospital or death with AKI. These datasets have been shown to be representative of patients across England and include data collected by many hundreds of GP practices. It is therefore expected that the findings are generalisable to the same population.^21^ A strength of this analysis was that it accounted for the competing risk of death in each analysis, which minimises the likelihood of overestimating the underlying risk of AKI. This is important for older patients, where the competing risk of death is high. The model only included predictors that are routinely available in primary care EHRs, and those predictors with missing data at implementation (such as alcohol consumption, ethnicity, and body mass index) could easily be collected within the patient consultation in which the tool is used.

This analysis had some limitations. First, model miscalibration was present, particularly for those with higher predicted risks, although this is common in prediction models based on EHRs commonly used in clinical practice,^22^ and has been observed in those previously developed for AKI.^23^ Such miscalibration would not be a problem in practice if using lower thresholds to define high/low risk (for example, +/–10% over 10 years).

Second, the model outcome was based on hospital and death registry codes, where AKI was listed as the primary cause of admission/death, rather than guideline recommended changes in creatinine,^5^ although many of these codes will have been based on creatinine measurements. This aimed to ensure that the AKI events were truly significant and hence meaningful for both patients and their GPs. It also avoided simply labelling individuals on the basis of blood results that may not have an impact on their quality of life,^24^ although the authors’ acknowledge that such test results are important and can lead to further nephrology referral, investigations, and medication changes, all of which can impose a burden on patients.

Finally, previous prediction models for AKI, based in a secondary care setting, have included conditions such as heart failure, respiratory failure, and prescription of non-steroidal anti-inflammatory medications.^23^^,^^25^ These predictors were not included in the present analysis and it is unclear whether their absence affected the present model performance on external validation.

### Comparison with existing literature

Previous clinical prediction models developed to predict AKI have almost exclusively focused on utility in an inpatient or post-operative setting,^9^^,^^23^^,^^26^ using data from a selected population of patients admitted to hospital for a range of conditions such as heart failure.^27^ These models estimated the risk of AKI over shorter periods of followup, did not account for the competing risk of death, and did not include prescribed medication as a potential predictor.

To the authors’ knowledge, this is the first clinical prediction model for AKI developed for use in a primary care setting and taking into account prescribed medication. Unlike many other models,^9^^,^^23^^,^^27^ the present model was externally validated in a nationally representative population and displayed better discrimination than previous models,^23^ even at 10-years post-index date.

### Implications for practice

The rationale for developing this model was to provide data to aid GPs in better understanding the balance of benefits and harms of antihypertensive therapy, before prescribing treatment or modifying existing prescriptions. To do this, GPs need to understand the effect of treatment on CVD^2^ and adverse events,^3^ and an individual’s underlying risk of benefit and harm. Many CVD prediction models exist that enable the benefits of antihypertensive treatment to be estimated,^4^ but unlike conditions such as atrial fibrillation where stroke prevention is routinely assessed against bleeding risk, the risk of harm from antihypertensive treatment is not well documented or understood.^3^^,^^28^^–^^30^ The present prediction model provides this information.

Given the low risk of AKI seen across the population, it seems likely that these particular harms of treatment would only outweigh the benefits in a small fraction of individuals. Indeed, in the present population, <1% of individuals had a high risk of AKI but low risk of CVD. These individuals were more likely to be obese, be from an area of high deprivation, or be prescribed multiple antihypertensive medications, but using the present tool alongside existing CVD prediction tools^4^ would provide the most personalised risk estimates. Such tools could also be enhanced by incorporating similar evidence regarding the risk of falls^31^ to develop a multidimensional antihypertensive harm tool.

Regular monitoring of creatinine levels is recommended in primary care, including in those with hypertension,^32^ and blood test results now routinely alert GPs to possible AKI.^6^ What GPs should do with this information remains unclear.^33^ The present prediction model could be used to target such monitoring to those most likely to benefit from it.

In conclusion, the present study developed and validated a clinical prediction model for admission to hospital or death with AKI, and found most patients with an indication for antihypertensives had a very-low risk of AKI. This model could be used to reassure patients starting or up-titrating antihypertensive treatment, and should be used alongside other prediction models for adverse events related to antihypertensive therapy^31^ to allow GPs and patients to better understand the full spectrum of benefits and harms from such treatment.

## Data

Data were obtained via a CPRD institutional licence. Requests for data sharing should be made directly to the CPRD. The algorithm is freely available for research use and can be downloaded from: https://process.innovation.ox.ac.uk/software (will be made publically available on publication of this manuscript). Codelists used to generate the study cohort and variables included in the analysis are available at: https://github.com/jamessheppard48/STRATIFY-BP/tree/STRATIFY-AKI.
